# Time to act: dismantling social barriers to healthy sleep across the life-course

**DOI:** 10.1093/eurpub/ckaf093

**Published:** 2025-07-31

**Authors:** Dayna A Johnson, Saverio Stranges

**Affiliations:** Department of Epidemiology, Rollins School of Public Health, Emory University, Atlanta, GA, United States; Department of Epidemiology and Biostatistics, Schulich School of Medicine & Dentistry, Western University, London, Ontario, Canada; Department of Family Medicine, Schulich School of Medicine & Dentistry, Western University, London, Ontario, Canada; Department of Medicine, Schulich School of Medicine & Dentistry, Western University, London, Ontario, Canada; Department of Clinical Medicine and Surgery, University of Naples Federico II, Naples, Italy

Poor sleep health is a recognized common problem across the life cycle globally. Macro-level factors contributing to this growing societal problem include the demands of a “Western” lifestyle, economic stressors, social, and environmental determinants, and global crises, including conflicts, political tensions, the COVID-19 pandemic and climate change. Approximately 30%–45% of adults report insufficient or poor-quality sleep, with prevalence rates varying significantly across demographic groups.

There is growing consensus that optimizing sleep health should be a public health priority because poor sleep patterns are modifiable risk factors for numerous health outcomes. Meta-analyses have linked inadequate sleep with increased risk of cardiovascular-related outcomes, cancer, cognitive decline, psychiatric illnesses, and premature mortality [1].

A consistent social gradient exists in the distribution of poor sleep health, as racial and ethnic minoritized groups, those with low socioeconomic status, migrants, refugees, indigenous people, unsheltered and those with disabilities are disproportionately affected [2, 3]. There is evidence that suggests these disparities in sleep are contributing to overall health disparities [4].

Importantly, sleep health trajectories fluctuate across critical life transitions, from infancy to late adulthood, due to several factors including socio-demographics, lifestyle behaviors, environmental contexts, physiological changes, and psychosocial stressors. This life-course perspective is essential for understanding how sleep patterns evolve and how social determinants differentially impact sleep health at various developmental stages.

Despite overwhelming evidence on sleep’s role in physical, mental, and social well-being, it remains absent from current public health priorities. In this viewpoint, we address social determinants of sleep health disparities across the life-course and propose policy initiatives to promote sleep health at the population level.

## Sleep disparities across the life-course

Sleep health disparities—differences in sleep health dimensions that adversely affect disadvantaged populations—are pervasive globally and manifest differently across life stages. These disparities align with the fundamental causes of health inequities theory [5], where social conditions determine sleep disparities through social stressors (discrimination, socioeconomic disadvantage) and adverse environmental factors (light exposure, neighborhood violence, air pollution).

During early childhood, inadequate sleep disproportionately affects children from disadvantaged backgrounds [6]. These early disparities are associated with behavioral problems, impaired learning, and developmental delays that can persist throughout childhood. Studies show that children from families with low socioeconomic status experience more fragmented sleep, shorter sleep duration, and more irregular bedtimes than children from higher socioeconomic backgrounds [6, 7].

Adolescence represents another critical period when biological sleep phase delay conflicts with early school start times. This biological shift affects all adolescents, but social factors exacerbate the impact for disadvantaged youth. Studies indicate adolescents in low socioeconomic environments have poorer sleep than those in more advantaged environments [8]. This disparity is partly explained by differences in home environments, technology use, and exposure to disruptive environmental factors.

In adulthood, sleep disparities persist even after controlling for behavioral and clinical factors, highlighting the influence of social determinants. Historically minoritized individuals disproportionately experience psychosocial stressors, which are associated with insufficient sleep. Adults from disadvantaged backgrounds are more likely to work multiple jobs or shift work, with shift workers having increased risk of developing sleep disorders alongside elevated cardiometabolic disease risks [9].

Environmental factors play a crucial role across all life stages. Disadvantaged individuals often reside in environments with higher nighttime noise and light pollution, hindering healthy sleep practices. Individuals in disadvantaged areas or rural areas may have a lack of access to sleep professionals and sleep-related care. Additionally, evidence identifies factors promoting healthy sleep across the life-course. Social support and positive neighborhood environments are associated with lower odds of sleep problems, potentially buffering stress effects on sleep regulation. These protective factors can be leveraged in public health strategies to mitigate stressors experienced by disadvantaged populations.

## Public health strategies and policy initiatives

Addressing sleep health disparities requires comprehensive approaches that consider both life-course stages and social determinants. Example initiatives include sleep health literacy programs in schools for children and adolescents, workplace programs for adults, and community-based awareness and screening programs. These programs should feature culturally tailored messaging that recognizes diverse sleeping arrangements, family structures, and cultural beliefs about sleep across different populations. Integration of sleep education into existing preventive healthcare services would ensure that vulnerable populations receive information at critical developmental periods.

Environmental interventions must address the disproportionate exposure to sleep-disrupting conditions in disadvantaged neighborhoods. Urban planning policies can reduce noise and light pollution, with priority given to areas with documented sleep health disparities. Housing standards that promote sleep-conducive environments, including adequate ventilation, temperature control, and sound insulation, would particularly benefit children and older adults who spend more time at home. Workplace policies limiting excessive shift work rotations and ensuring adequate recovery periods between shifts could significantly reduce sleep disparities. Flexible work schedules accommodating individual chronotypes would benefit parents of young children and caregivers, roles disproportionately affecting sleep health in disadvantaged communities.

Healthcare systems can address sleep disparities through provider training on culturally competent approaches to sleep problems and integration of sleep health screening into routine primary care visits. Improving accessibility of sleep disorder diagnosis and treatment for underserved populations is particularly important for older adults who experience multiple sleep disorders. Economic strategies addressing poverty through living wage policies and income support programs would indirectly benefit sleep by reducing stress and improving housing options for families with children, while social support programs for vulnerable populations could build on evidence that social connections buffer sleep disruption.

Optimizing sleep health across the life span should be a public health priority. A life-course perspective reveals how sleep health disparities begin in childhood, accumulate through adulthood, and manifest as significant health inequities in later life. Given sleep’s multidimensional nature, there is a need for multisectoral approaches tackling macro-level factors contributing to sleep health trajectories across the life-course.

The burden of poor sleep disproportionately affects disadvantaged populations, making sleep health a social justice issue. Addressing social determinants through policy interventions targeting housing quality, neighborhood pollution, and shift work regulations, coupled with culturally tailored awareness campaigns, would help reduce sleep disparities across the life course.

Public health approaches must move beyond individual-level interventions to address structural factors shaping sleep opportunities from childhood through older adulthood. By implementing comprehensive strategies that recognize how social contexts shape sleep across different life stages, we can work toward making healthy sleep accessible to all, regardless of social position, contributing to reducing broader health inequities affecting vulnerable communities.

Conflict of interest: None declared.

## Data Availability

Not applicable.
